# Septic Arthritis and Related Conditions

**DOI:** 10.3390/children9050751

**Published:** 2022-05-19

**Authors:** Teresa Giani, Rolando Cimaz

**Affiliations:** Department of Clinical Sciences and Community Health, Research Center for Adult and Pediatric Rheumatic Diseases, University of Milan, 20112 Milan, Italy

Arthritis is a common condition that any pediatrician may have to deal with. Its symptoms include joint swelling, tenderness, and decreased range of motion, although in some situations and in very young children, clinical signs may not be clearly defined.

Arthritis may involve one, few, or many joints; it may show an abrupt and very symptomatic onset or a mild presentation, may have a relatively brief duration or a prolonged course, can exhibit an additive or a migratory pattern, and may be induced by several different causes such as mechanic, autoimmune, infectious, neoplastic, metabolic, or genetic origins, all requiring different types of management. At the onset, it is hard to predict the disease course and to discern between different etiologies, so it is crucial to proceed with a work-up program combining laboratory, instrumental, and clinical data in order to first rule out those conditions associated with serious or even life-threatening consequences such as infections or neoplasia.

In this Special Issue, Gamalero et al. summarized the main characteristics of septic arthritis, defining the differences with other articular conditions [1]. Septic arthritis refers to an infection of the joint space; it occurs more commonly in children than in adults, with a peak in the first few years of life. This condition is responsible for more than fifty percent of acute arthritides in preschoolers and is associated with a significant morbidity and mortality rate. Local, systemic, and laboratory signs of inflammation are usually evident, and often, only one joint is involved, which is commonly a large joint of the lower limbs. However, features may vary with the age and the immunocompetence of the host, the affected joint, and the pathogen. Some microorganisms, such as *Kingella kingae*, or a reduced immune response may be responsible for indolent clinical presentation as well as multiple-joint infection.

The most common causative agents are bacteria, and their specific involvement changes on the basis of the age of the patient, underlying medical conditions, and geographic area. An increase in peripheral white blood cells, erythrocyte sedimentation rate (ESR), and C-reactive protein (CRP) can be observed in the early stages and can support the initiation of empiric intravenous (IV) antibiotics. However, the combination of blood and synovial fluid cultures and polymerase chain reaction analyses conclusively confirms the diagnosis and allows the choice of targeted antimicrobial therapy. Imaging techniques are useful to exclude the presence a concomitant infection of bone and surrounding tissues, as well as other underlying conditions such as fractures.

As described by Di Pietro et al., septic arthritis should always be suspected in any young child with a new-onset painful joint, especially when associated with local inflammatory signs and increased laboratory inflammatory markers [2]. Indeed, prompt empiric IV antimicrobial therapy can avoid the extension of the infection to adjacent bones (as the authors described in their young patient) and prevent permanent joint impairments; once cultures are used to identify the causative agent, a broad-spectrum antibiotic regimen can be changed to a targeted one. However, in almost half of the analyses, microbiological diagnosis fails to identify the pathogen, or, as in the case of the young girl described by Di Pietro et al., even the susceptibility test may not provide useful results. In these cases, treatment should be driven by the clinical course, also considering patient’s age, immune status, concomitant diseases, and epidemiological data.

Although septic arthritis needs to be ruled out first in a child with new-onset arthritis, especially when the case is monoarticular, Gamalero et al. also underlined the importance of an exhaustive differential diagnosis and described the common causes of arthritis in the pediatric age, offering an overview of the clinical, laboratory, and radiological features most useful in distinguishing among different etiologies and proposing a practical flow-chart for use in diagnosis [1]. The authors informed readers how, in pediatric-age patients, neoplastic conditions, especially malignant bone tumors and lymphoproliferative disorders, may be responsible for arthropathy and musculoskeletal symptoms and, even if histological confirmation is necessary, simple laboratory tests and/or imaging can be useful in the early stages [1].

Orthopedic disorders are usually characterized by pain with mechanical characteristics (with motion and not after rest) and by good general conditions. Reactive arthritis shows a self-limiting course, and usually, only symptomatic treatments are required, but at the onset of the disease, it can hardly be distinguished from septic arthritis. The authors suggested searching for laboratory evidence of a recent group A β-hemolytic streptococcal infection in order to exclude rheumatic fever that, contrary to other types of reactive arthritides, may be complicated by unnoticed cardiac damage.

Among the most difficult differential diagnoses is chronic nonbacterial osteomyelitis (CNO). First described in 1972, it is a rare, non-infectious autoinflammatory bone disease that typically presents in patients of pediatric age and is characterized by relapsing and remitting episodes of bone pain [3,4]. Almost 40% of patients also develop arthritis, and sometimes, children show increased inflammatory markers that can make clinicians to consider the possibility of an infection such as osteomyelitis or septic arthritis. A biopsy is sometimes necessary, as CNO is diagnosed through exclusion.

In their article, Koryllou et al. described clinical manifestations, laboratory and radiological features, treatment options, and known pathophysiological mechanisms related to CNO, also proposing a schematic decisional algorithm for children complaining of unifocal/multifocal osteoarticular pain [4].

Finally, the issue also considers juvenile idiopathic arthritis (JIA), the most common chronic rheumatic disease in childhood. This condition has a chronic and paucisymptomatic course with normal inflammatory markers, especially in those forms with few joints involved. However, both septic arthritis and JIA, especially the oligoarticular subtype, predominate in the first few years of life when infections may show a mild and subtle expression, thus making it difficult to distinguish between these two conditions at the onset of the conditions. A delay in septic arthritis diagnosis may lead to potential permanent joint sequelae, while a delay in JIA diagnosis not only predisposes the individual to articular complications but also can defer uveitis identification. Indeed, more than a quarter of JIA patients develop uveitis that is usually silent, with a chronic course and a significant risk of developing sight-threatening complications [5]. Thus, an ophthalmologic screening is paramount in children in which JIA is suspected. In their review, Carlsson et al. offered a summary regarding the main clinical and epidemiological characteristics of JIA-associated uveitis, illustrated recent, innovative risk factor analyses such as genomic, transcriptomic, and proteomic studies, and described the current therapeutic strategies [6].

In conclusion, septic arthritis represents one of the most frequent causes of arthritis in pediatric-age patients and is associated with a high mortality and morbidity risk; thus, it must receive immediate consideration. However, many other types of arthritis, requiring different managements, may affect children, and a selective work-up based on clinical signs and supported by laboratory and imaging data should motivate clinicians.
